# Glycogen Synthase Kinase-3β and Caspase-2 Mediate Ceramide- and Etoposide-Induced Apoptosis by Regulating the Lysosomal-Mitochondrial Axis

**DOI:** 10.1371/journal.pone.0145460

**Published:** 2016-01-04

**Authors:** Chiou-Feng Lin, Cheng-Chieh Tsai, Wei-Ching Huang, Yu-Chih Wang, Po-Chun Tseng, Tsung-Ting Tsai, Chia-Ling Chen

**Affiliations:** 1 Department of Microbiology and Immunology, School of Medicine, College of Medicine, Taipei Medical University, Taipei, 110, Taiwan; 2 Graduate Institute of Medical Sciences, College of Medicine, Taipei Medical University, Taipei, 110, Taiwan; 3 Department of Nursing, Chung Hwa University of Medical Technology, Tainan, 717, Taiwan; 4 Institute of Clinical Medicine, College of Medicine, National Cheng Kung University, Tainan, 701, Taiwan; 5 Translational Research Center, Taipei Medical University, Taipei, 110, Taiwan; Karolinska Institutet, SWEDEN

## Abstract

Glycogen synthase kinase-3β (GSK-3β) regulates the sequential activation of caspase-2 and caspase-8 before mitochondrial apoptosis. Here, we report the regulation of Mcl-1 destabilization and cathepsin D-regulated caspase-8 activation by GSK-3β and caspase-2. Treatment with either the ceramide analogue C_2_-ceramide or the topoisomerase II inhibitor etoposide sequentially induced lysosomal membrane permeabilization (LMP), the reduction of mitochondrial transmembrane potential, and apoptosis. Following LMP, cathepsin D translocated from lysosomes to the cytoplasm, whereas inhibiting cathepsin D blocked mitochondrial apoptosis. Furthermore, cathepsin D caused the activation of caspase-8 but not caspase-2. Inhibiting GSK-3β and caspase-2 blocked Mcl-1 destabilization, LMP, cathepsin D re-localization, caspase-8 activation, and mitochondrial apoptosis. Expression of Mcl-1 was localized to the lysosomes, and forced expression of Mcl-1 prevented apoptotic signaling via the lysosomal-mitochondrial pathway. These results demonstrate the importance of GSK-3β and caspase-2 in ceramide- and etoposide-induced apoptosis through mechanisms involving Mcl-1 destabilization and the lysosomal-mitochondrial axis.

## Introduction

Lysosomal apoptosis is generally caused by oxidative stress, tumor necrosis factor-α (TNF-α), staurosporine, and anticancer agents [1–3]. A variety of intracellular stimuli, including calcium, reactive oxygen species, ceramide, sphingosine, phospholipases, and caspases, lead to lysosomal membrane permeabilization (LMP) [2–5]. Additionally, pro-apoptotic Bax, Bid, and Bim can be translocated to lysosomes and cause LMP in TNF-α-, TNF-α-related apoptosis-inducing ligand (TRAIL)-, and free fatty acid-induced apoptosis [5–7]. Following LMP, cytosolic acidification occurs and cathepsins translocate from lysosomes to the cytoplasm [1,2,8]. The levels of LMP and cytosolic acidification determine whether the cell will undergo apoptosis or apoptotic-like necrosis [2]. Cathepsin B/L or cathepsin D causes Bax and Bid activation followed by mitochondrial apoptosis [8]. Furthermore, these proteins are able to cause caspase-2 and caspase-8 activation before mitochondrial damage [9,10]. The involvement of the lysosomal-mitochondrial axis is therefore speculated in at least some forms of apoptosis [3,8–10].

Glycogen synthase kinase-3β (GSK-3β), a serine/threonine kinase, is now known to be important for ceramide-, staurosporine-, heat shock-, growth factor withdrawal-, hypoxia-, and endoplasmic reticulum stress-induced apoptosis [11]. For GSK-3β activation, protein phosphatase 2A (PP2A) may concomitantly dephosphorylate and activate it directly or indirectly by downregulating Akt, a negative regulator of GSK-3β [11–13]. Active GSK-3β phosphorylates its downstream substrates, such as translation initiation factor 2B, β-catenin, p21^Cip1^, p53, and Bax, which are required for determining cell fate [14]. In addition, GSK-3β causes Mcl-1 phosphorylation followed by destabilization or degradation via the ubiquitin-proteasome system [15,16]. Caspases such as caspase-9 and caspase-3 also cause Mcl-1 destabilization in UV-, irradiation-, etoposide-, and TRAIL-induced apoptosis [17–19].

The generation of ceramide is involved in apoptotic stimulation by TNF-α, CD95, serum withdrawal, chemotherapeutic agents, and irradiation [20–22]. We and others demonstrated that ceramide induces caspase-2 and caspase-8 activation followed by Bid activation upstream of mitochondrial damage [23–25]. We further demonstrated that GSK-3β knockdown using short interfering RNA or specific inhibitors renders cells resistant to ceramide-induced caspase-2 and caspase-8 activation and mitochondrial apoptosis [11]. However, the mechanism of the sequential activation of caspase-2 and caspase-8 remains unresolved. In the present study, we show that GSK-3β and caspase-2 cause Mcl-1 destabilization followed by LMP and cathepsin D-mediated caspase-8 activation in lysosomal-mitochondrial axis-mediated apoptosis induced by either the ceramide analogue C_2_-ceramide or the topoisomerase II inhibitor etoposide.

## Materials and Methods

### Cells, culture condition, and reagents

The mouse T hybridoma cell line 10I was kindly provided by Dr. M. Z. Lai, Academia Sinica, Taiwan [24,26]. 10I cells were cultured in RPMI 1640 medium supplemented with 10% heat-inactivated fetal bovine serum, 50 units/ml penicillin, and 0.05 mg/ml streptomycin. Human lung epithelial A549 cells (CCL185, ATCC) were cultured in DMEM supplemented with 10% heat-inactivated fetal bovine serum, 50 units/ml penicillin, and 0.05 mg/ml streptomycin. All cells were maintained at 37°C in 5% CO_2_. Mcl-1-overexpressing A549 cells were constructed using the transfection reagent LipofectAMINE 2000; Invitrogen, Carlsbad, CA) and then cultured in RPMI supplemented with 10% fetal bovine serum. pcDNA3-HA and pcDNA3-HA-hMcl-1 were a gift from Dr. Yu-Ming Wang, National Cheng Kung University, Tainan, Taiwan, and Dr. Hsin-Fang Yang-Yen, Academia Sinica, Taipei, Taiwan, and were prepared as described previously [27]. The ceramide analogues C_2_-ceramide and C_2_-dihydroceramide and the topoisomerase II inhibitor etoposide (Sigma-Aldrich, St. Louis, MO) were dissolved in dimethyl sulfoxide (DMSO). The broad-spectrum caspase inhibitor benzyloxycarbonyl-Val-Ala-Asp(O-Me)-fluoro methyl ketone (z-VAD-fmk) and the caspase-8 and caspase-2 inhibitors benzyloxycarbonyl-Ile-Glu(O-Me)-Thr-Asp(O-Me)-fluoromethyl ketone (z-IETD-fmk) and benzyloxycarbonyl-Val-Asp(O-Me)-Val-Ala-Asp(O-Me)-fluoromethyl ketone (z-VDVAD-fmk), respectively, were purchased from Sigma-Aldrich and dissolved in DMSO. The cathepsin B inhibitor benzyloxycarbonyl-Phe-Ala-fluoromethyl ketone (z-FA-fmk) was purchased from Calbiochem (San Diego, CA) and dissolved in DMSO. The cathepsin D inhibitor pepstatin A (Sigma-Aldrich) was dissolved in methanol containing 10% ethanol. The GSK-3β inhibitor lithium chloride (LiCl; Sigma-Aldrich), the PP2A inhibitor okadaic acid (OA; Sigma-Aldrich), and the proteasome inhibitor MG132 (Tocris, Bristol, UK) were dissolved in DMSO. Purified human cathepsin D was purchased from Sigma-Aldrich.

### Lysosomal membrane permeabilization (LMP) assay

For LMP assay, cells were treated with 5 μg/ml of acridine orange (AO; Sigma-Aldrich) in serum-free RPMI or DMEM for 15 min at 37°C. After being washed with phosphate-buffered saline (PBS) twice, a flow cytometer (FACSCalibur; BD Biosciences, San Jose, CA) was used in FL-3 channel (>650 nm) following excitation at 488 nm. Meanwhile, cells were observed using a laser scanning confocal microscope (Leica TCS SPII, Nussloch, Germany) equipped with an argon ion laser (488 and 514 nm).

### Mitochondrial function assay

For the detection of the loss of mitochondrial transmembrane potential (MTP), cells were incubated with 50 μM rhodamine 123 (Sigma-Aldrich) in culture medium for 30 min at 37°C. After being washed with PBS twice, cells were analyzed by using a flow cytometer (FACSCalibur) in FL-1 channel (515–545 nm) following excitation at 488 nm.

### Analysis of cell apoptosis

Cell apoptosis characterized by DNA fragmentation was detected using propidium iodide (PI; Sigma-Aldrich) staining. After fixation with 70% ethanol in PBS, cells were stained with PI/RNase working solution in PBS containing 40 μg/ml PI and 100 μg/ml RNase A (Sigma-Aldrich) for 30 min at room temperature and then analyzed using flow cytometry (FACSCalibur) with excitation at 488 nm and emission detected in the FL-2 channel (564–606 nm). Samples were analyzed using CellQuest Pro version 4.0.2 software (BD Biosciences), and quantification was carried out with WinMDI version 2.8 software (The Scripps Institute, La Jolla, CA). Small cell debris was excluded by gating for forward scatter. To validate the quantification of apoptotic cells from flow cytometric analysis, cell apoptosis was also detected using 4,6-diamidino-2-phenylindole (DAPI; Sigma-Aldrich) staining at a concentration of 5 μg/ml at room temperature for 10 min. After being washed with PBS, the cells were visualized under a fluorescent microscope (IX71, Olympus, Japan).

### Immunostaining

Cells were fixed with 1% formaldehyde in PBS and permeabilized with 0.01% saponin in PBS. Rabbit polyclonal antibodies against human cathepsin D were used, followed by Alexa 488-conjugated goat anti-rabbit IgG (Invitrogen) staining. Cells were observed using a laser scanning confocal microscope (Leica TCS SPII). DAPI (Invitrogen) was used for nuclear staining.

### Short interfering RNA (siRNA) and lentiviral-based short hairpin RNA (shRNA) transfection

Human cathepsin D (sc-29239) was silenced using a commercial siRNA kit (Santa Cruz Biotechnology, Santa Cruz, CA). Transfection was performed by electroporation using a Microporator (Digital Bio Technology, Korea). Before transfection, cells were washed with serum-free DMEM and mixed with siRNA (50 nM) in Opti-MEM medium (Invitrogen) in a volume of 100 μl. After transfection, cells were incubated for 48 h in DMEM supplemented with 10% fetal bovine serum at 37°C prior to stimulation. A non-specific scrambled siRNA (sc-37007, Santa Cruz Biotechnology) was used as the negative control. Lentiviral-based caspase-2 and GSK-3β knockdown in 10I cells was performed. shRNA clones were obtained from the National RNAi Core Facility, located at the Institute of Molecular Biology/Genomic Research Center, Academia Sinica, Taiwan. The mouse library should be referred to as TRC-Mm 1.0. The constructs that were used in 10I cells (shRNA target sequence TRCN0000012613 5’-CGAGAAGAAAGATGAGGTCTA-3’ for mouse GSK-3β-1; TRCN0000012614 5’-CCACTCAAGAACTGTCAAGTA-3’ for mouse GSK-3β-2; TRCN0000012615 5’-CATGAAAGTTAGCAGAGATAA-3’ for mouse GSK-3β-3; TRCN0000012616 5’-CCACAGAACCTCTTGTTGGAT-3’ for mouse GSK-3β-4; TRCN0000012617 5’-CGGGACCCAAATGTCAAACTA-3’ for mouse GSK-3β-5; TRCN0000012238 5’-CCCAATTTAATGTAGGTGTTT-3’ for mouse caspase-2-1; TRCN0000012239 5’-CCTCCTAGAGAAGGACATTAT-3’ for mouse caspase-2-2; TRCN0000012240 5’-GCTACGGAACACTCCTTAGAT-3’ for mouse caspase-2-3; TRCN0000012241 5’-GCCCTTATCAAGGAGCGTGAA-3’ for mouse caspase-2-4; TRCN0000012242 5’-CCTTAAAGGTAATGCTGCCAT-3’ for mouse caspase-2-5) were used to generate recombinant lentiviral particles. Recombinant lentivirus were prepared according to our previous study [11]. After transduction for 24 h, protein expression was monitored using Western blot analysis.

### Western blot analysis

Total cell extracts were separated by SDS-PAGE and then transferred to a polyvinylidene difluoride membrane (Millipore Corporation, Billerica, MA). After blocking, blots were developed with a series of antibodies as indicated. Rabbit antibodies specific for mouse and human caspase-2, -3, -8, poly(ADP-ribose) polymerase (PARP) (Cell Signaling Technology, Beverly, MA), Bid, and tBid (Oncogene, San Diego, CA) were used. Monoclonal antibodies against β-actin and LAMP-1 (Sigma-Aldrich) and rabbit antibodies against mouse and human cathepsin D, Mcl-1, and COX IV (Cell Signaling Technology) and rabbit anti- GSK-3β (Santa Cruz Biotechnology) were used. Finally, blots were hybridized with horseradish peroxidase-conjugated goat anti-rabbit IgG or anti-mouse IgG (Calbiochem) and developed using enhanced chemiluminescence (Pierce, Rockford, IL).

### Detection of caspase-8 activity

Cellular caspase activation was determined using a caspase-2 and caspase-8 assay kit (Calbiochem) according to the manufacturer’s instructions. Optical density (OD) measurements were performed using a Spectra MAX 340PC microplate reader (Molecular Devices, Sunnyvale, CA).

### *In vitro* cathepsin D-mediated caspase-8 activation

Total cell lysates were extracted with lysis buffer containing 1% Triton X-100, 50 mM Tris (pH 7.5), 10 mM EDTA, and 0.02% NaN_3_ (Roche Boehringer Mannheim Diagnostics, Mannheim, Germany). For the detection of caspase-8 activation, cell lysates (100 μg), diluted with reaction buffer containing 330 mM sucrose, 2 mM HEPES, 5 mM MgCl_2_ (pH 7.4), were incubated with or without 0.5 U of purified cathepsin D (Sigma-Aldrich) for 0.5 h at 37°C. The activation of caspase-8 was determined by activity assay and Western blotting.

### Purification of lysosomes

Lysosomes were isolated using a Lysosome Isolation Kit (Sigma-Aldrich) according to the manufacturer’s instructions. Cells equaling 1 ml of packed cell volume were homogenized in extraction buffer (0.25 M sucrose, 20 mM HEPES/KOH, 1 mM EDTA, 2 mM MgCl_2_, and 10 mM KCl) with a Dounce homogenizer. The homogenate was centrifuged at 1,000×*g* for 10 min, and the resulting supernatant was centrifuged at 20,000×*g* for 20 min to yield the crude lysosomal fraction (pellet). To further enrich the lysosomes, the crude lysosomal fraction was suspended and diluted with 2.3 M sucrose to a 19% gradient fraction and then loaded on an assembled sucrose gradient (27>22.5>19>16>12>8%) followed by centrifugation at 45,000×*g* for 3 h without braking. Several bands formed after centrifugation, and the top band, which contained the isolated lysosomes, was carefully removed and saved on ice for further examination.

### Statistical analysis

Student’s two-tailed unpaired *t* test or one-way ANOVA analysis followed by Dunnet post-hoc test, as appropriate with commercially available statistical software (SigmaPlot 8.0 for Windows; Systat Software, Inc., San Jose, CA) were performed. Values are expressed as means ± S.D. and a *P*-value of 0.05 was considered statistically significant.

## Results

### Ceramide or etoposide induces LMP, MTP reduction, and apoptosis

**Fig 1:**
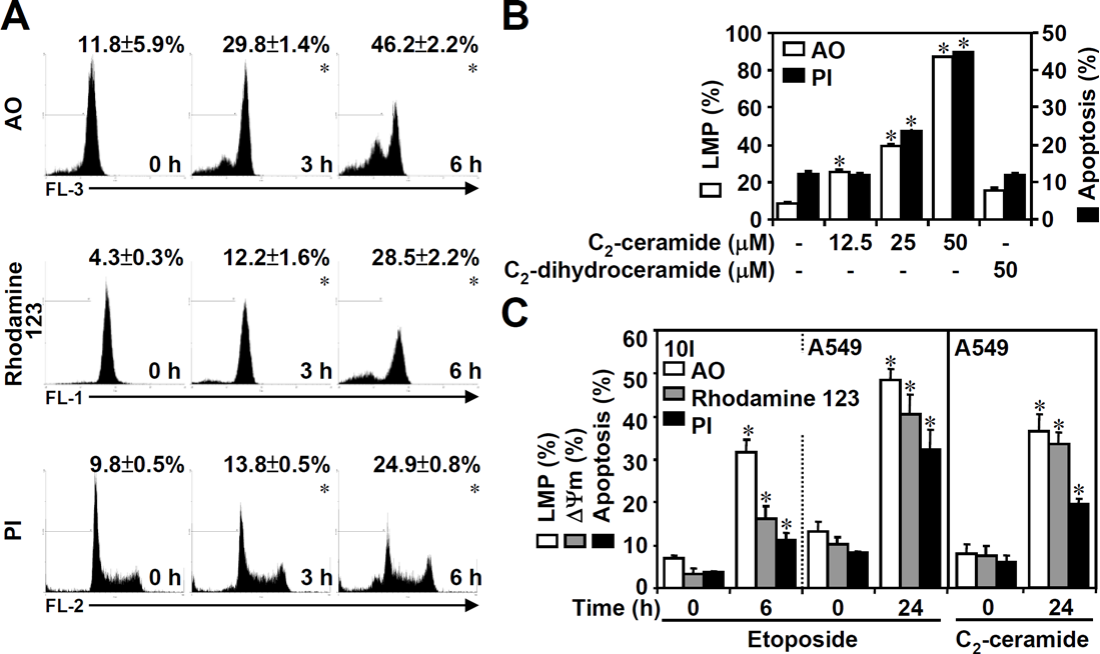
Ceramide or etoposide causes lysosomal destabilization and mitochondrial apoptosis. Mouse T hybridoma 10I cells were treated with 25 μM C_2_-ceramide for the indicated time periods (A) or with different doses of C_2_-ceramide (B) for 6 h. 10I cells and human epithelial A549 cells were treated with 25 μM C_2_-ceramide or 50 μM etoposide for the indicated time periods (C). Using AO (top), rhodamine 123 (middle), and PI (bottom) staining, respectively, followed by flow cytometric analysis, the percentages of cells with lysosomal membrane permeabilization (LMP), the reduction of MTP (ΔΨ_m_), and apoptosis are shown (means ± S.D. of three individual experiments). C_2_-dihydroceramide was used as a negative control. A representative histogram obtained from 10I cells and shown in A. *, *P* < 0.05 compared with the untreated group.

We used the ceramide analogue C_2_-ceramide (25 μM) or the topoisomerase II inhibitor etoposide (50 μM) to induce apoptosis in mouse 10I T hybridoma cells and human A549 lung epithelial carcinoma cells. The kinetics (Fig 1A top) and dose responses (Fig 1B) of C_2_-ceramide-induced LMP in 10I cells were shown by staining with AO, a monomeric cationic fluorescent dye, followed by flow cytometric analysis. By staining with the lipophilic cationic fluorochrome rhodamine 123, we found that C_2_-ceramide induced a time-dependent MTP reduction (Fig 1A middle) in 10I cells. Furthermore, PI staining followed by flow cytometric analysis demonstrated that C_2_-ceramide caused 10I cell apoptosis in a time- (Fig 1A, bottom) and dose-dependent manner (Fig 1B). However, C_2_-dihydroceramide, a stereoisomer of ceramide, did not cause LMP or apoptosis in 10I cells (Fig 1B). Further results confirmed that either C_2_-ceramide or etoposide caused LMP, MTP reduction, and apoptosis in A549 cells (Fig 1C). These results show that either ceramide or etoposide can induce lysosomal destabilization and mitochondrial apoptosis.

### Ceramide or etoposide induces mitochondrial apoptosis in a lysosomal cathepsin D-regulated manner

**Fig 2:**
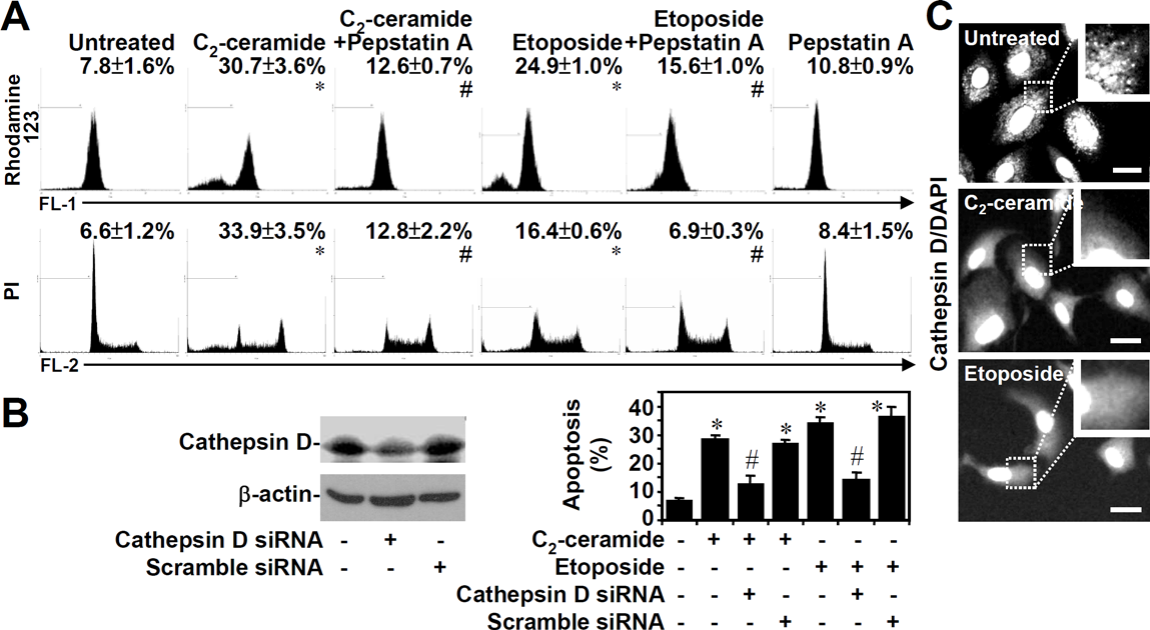
Ceramide or etoposide causes cathepsin D translocation followed by mitochondrial apoptosis. (A) 10I cell MTP reduction and apoptosis induced by 25 μM C_2_-ceramide or 50 μM etoposide for 6 h were determined in the presence or absence of the cathepsin D inhibitor pepstatin A (25 μM) using rhodamine 123 (top) and PI (bottom) staining, respectively, followed by flow cytometric analysis (means ± S.D. of three individual experiments). A representative histogram is shown. *, *P* < 0.05 compared with the untreated group. #, *P* < 0.05 compared with the ceramide-treated or etoposide-treated group. (B) Blockage of ceramide- or etoposide-induced cell apoptosis by cathepsin D siRNA. A549 cells were transfected with siRNA (50 nM) against cathepsin D or a scrambled control. Cathepsin D expression was detected by Western blotting (left). β-actin served as an internal control. Transfected cells were treated with ceramide (25 μM) or etoposide (50 μM) for 24 h and stained with PI followed by flow cytometric analysis (right). The percentages of apoptotic cells are shown (means ± S.D. of three individual experiments). *, *P* < 0.05 compared with the untreated group. #, *P* < 0.05 compared with the ceramide-treated or etoposide-treated group. (C) A549 cells were treated with 25 μM C_2_-ceramide or 50 μM etoposide for 24 h. After fixation, cells were incubated with a cathepsin D-specific antibody followed by an Alexa Fluor 488-labeled secondary antibody and DAPI nuclear staining. Cathepsin D translocation from lysosomes (characterized by punctate staining) to the cytoplasm (characterized by diffuse staining) is shown. The scale bar is 10 μm.

Lysosomes contain numerous proteases, such as cathepsin B/L and cathepsin D, which are activated and released following LMP [1,2,8]. To examine the potential roles of lysosomal cathepsins on ceramide (25 μM)- or etoposide (50 μM)-induced mitochondrial apoptosis, by using rhodamine 123 and PI staining, respectively, we found that the cathepsin D inhibitor pepstatin A (25 μM) blocked both C_2_-ceramide- and etoposide-induced MTP reduction (Fig 2A, top) as well as apoptosis (Fig 2A, bottom). To further confirm these results, we introduced siRNA specific for cathepsin D into A549 cells to block cathepsin D expression. The inhibition of cathepsin D expression was observed in cathepsin D siRNA (50 nM)-expressing cells but not in scramble control cells (Fig 2B, left). Both C_2_-ceramide- and etoposide-induced A549 cell apoptosis were blocked in cathepsin D siRNA-transfected cells, as determined by PI staining followed by flow cytometric analysis (Fig 2B, right). We next investigated whether ceramide or etoposide caused the release of cathepsin D from lysosomes to the cytoplasm. Using a cathepsin D-specific antibody, either C_2_-ceramide or etoposide caused cathepsin D re-localization (Fig 2C) from lysosomes (characterized by punctate staining) to the cytoplasm (characterized by diffuse staining). These results indicate that either ceramide or etoposide can induce LMP, followed by cathepsin D-regulated mitochondrial apoptosis.

### Ceramide or etoposide induces cathepsin D-regulated caspase-8 but not caspase-2 activation followed by mitochondrial apoptosis

**Fig 3:**
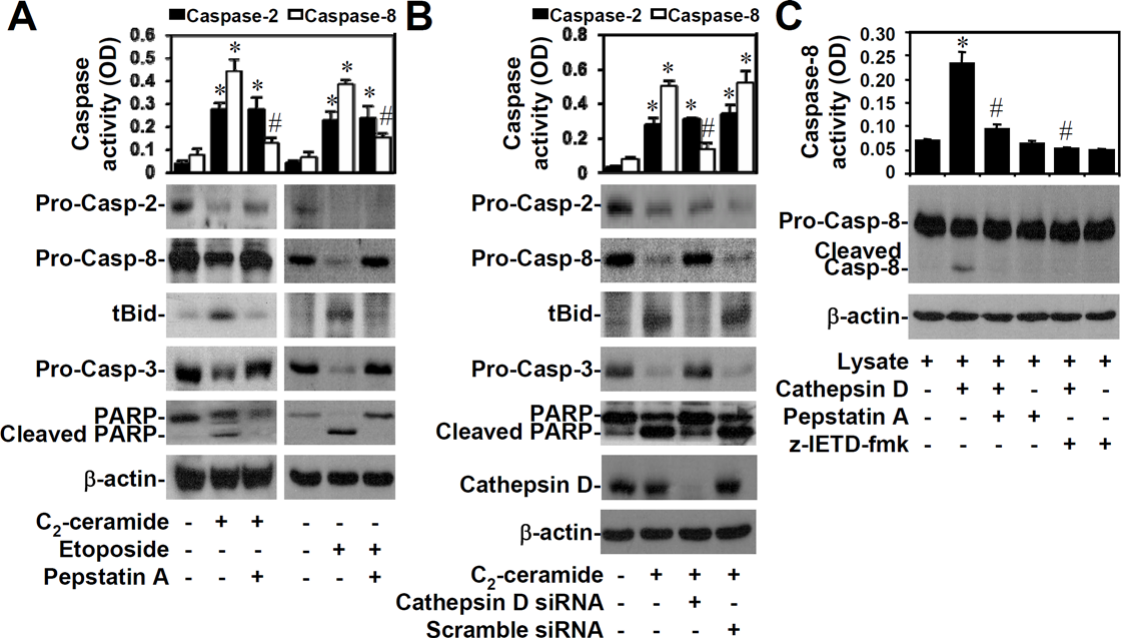
Inhibiting cathepsin D inhibits the ceramide- or etoposide-induced activation of caspase-8, but not caspase-2, and mitochondrial apoptosis. (A) 10I cells were treated with 25 μM C_2_-ceramide or 50 μM etoposide for 6 h in the presence or absence of the cathepsin D inhibitor pepstatin A (25 μM). (B) A549 cells were treated with 25 μM C_2_-ceramide for 24 h with or without cathepsin D siRNA (50 nM) pretreatment. (C) To further determine whether cathepsin D affects caspase-8 activation, recombinant human cathepsin D was incubated together with lysates from untreated A549 cells for 0.5 h at 37°C with or without 25 μM pepstatin A or 10 μM of the caspase-8 inhibitor z-IETD-fmk. We used enzymatic cleavage of the specific substrates benzyloxycarbonyl-Val-Asp(-OMe)-Val-Ala-Asp(-OMe)-pNA and benzyloxycarbonyl-Ile-Glu(-OMe)-Thr-Asp(-OMe)-pNA to determine the activities of caspase-2 and caspase-8, respectively. OD, optical density. Data are given as the average of three individual experiments (means ± S.D.). *, *P* < 0.05 compared with the untreated group. #, *P* < 0.05 compared with ceramide-treated, etoposide-treated, or cathepsin D-treated group. We used Western blotting to determine the activation of caspase-2, caspase-8, truncated Bid (tBid), caspase-3, PARP, and cathepsin D. β-actin served as an internal control.

We previously demonstrated that ceramide caused the sequential activation of caspase-2 and caspase-8 upstream of mitochondrial apoptosis [24]. We next examined the association of cathepsin D inhibition with defective initiator caspase activation. We first inactivated cathepsin D in 10I cells by pretreatment with pepstatin A (25 μM). By using caspase activity detection and Western blot analysis, the results showed that pretreatment with pepstatin A suppressed the activation of caspase-8, Bid, and caspase-3, as well as PARP cleavage, but not caspase-2, in response to either C_2_-ceramide (25 μM) (Fig 3A, left) or etoposide (50 μM) (Fig 3A, right) stimulation. Further confirming these results, cathepsin D siRNA (50 nM)-treated cells were defective in the C_2_-ceramide-induced activation of caspase-8, Bid, caspase-3, and PARP compared to the scramble control. However, cathepsin D siRNA did not inhibit caspase-2 activation (Fig 3B). We then investigated whether cathepsin D caused caspase-8 activation *in vitro*. Caspase-8 activity assays and Western blot analyses demonstrated that cathepsin D caused caspase-8 activation (Fig 3C). Therefore, cathepsin D is required for either ceramide or etoposide to induce the activation of caspase-8, but not caspase-2, and subsequent apoptotic signals of mitochondrial damage.

### Inhibiting GSK-3β or caspase-2 reduces ceramide-induced LMP, cathepsin D re-localization, and cell apoptosis

**Fig 4:**
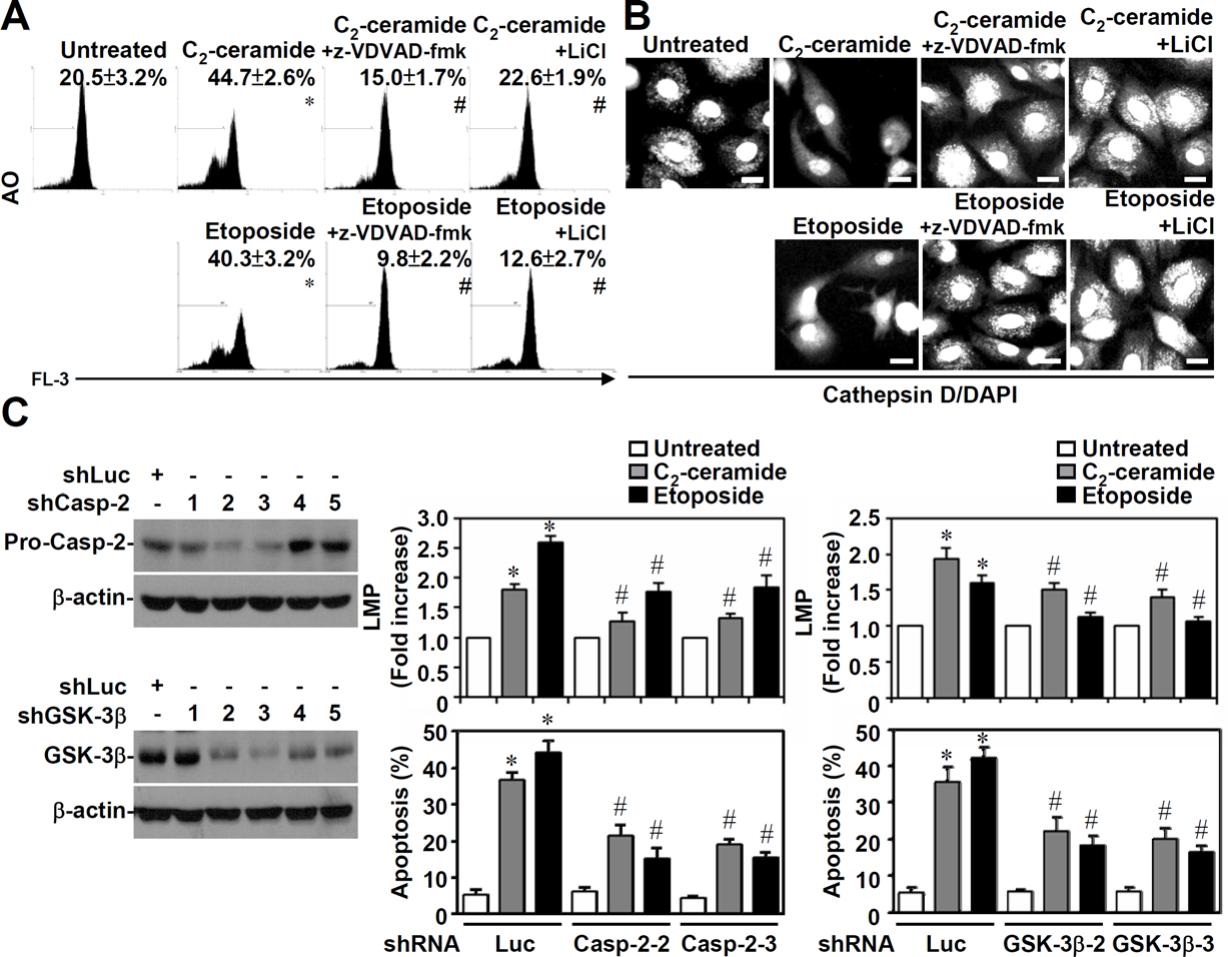
Inhibiting caspase-2 or GSK-3β blocks ceramide- or etoposide-induced LMP and cathepsin D translocation. (A) 10I cells were treated with 25 μM C_2_-ceramide or 50 μM etoposide for 6 h in the presence or absence of 10 μM of the caspase-2 inhibitor z-VDVAD-fmk or 10 mM of the GSK-3β inhibitor lithium chloride (LiCl). Using AO staining followed by flow cytometric analysis, the percentages of cells with LMP are shown (means ± S.D. of three individual experiments). A representative histogram is shown. *, *P* < 0.05 compared with the untreated group. #, *P* < 0.05 compared with the ceramide-treated or etoposide-treated group. (B) The translocation of cathepsin D in 25 μM C_2_-ceramide- or 50 μM etoposide-treated A549 cells for 24 h was determined in the presence or absence of 10 μM z-VDVAD-fmk or 10 mM LiCl using a cathepsin D-specific antibody followed by an Alexa Fluor 488-labeled secondary antibody and DAPI nuclear staining. The scale bar is 10 μm. (C) Blockage of ceramide- or etoposide-induced LMP and apoptosis by knockdown of caspase-2 or GSK-3β expression. 10I cells were transfected with shRNAs (five clones in total) against caspase-2 (shCasp-2) or GSK-3β (shGSK-3β) or a negative-control shRNA (shLuc). Caspase-2 and GSK-3β expression was detected by Western blotting. β-actin served as an internal control. Ceramide (25 μM)- or etoposide (50 μM)-treated cells transfected with effective clones as indicated for 6 h were stained with AO or PI followed by flow cytometric analysis. The fold-increase of cells with LMP and the percentages of apoptotic cells are shown (means ± S.D. of three individual experiments). *, *P* < 0.05 compared with the untreated group. #, *P* < 0.05 compared with the ceramide-treated shLuc or etoposide-treated shLuc group.

We previously demonstrated that GSK-3β activation was essential for ceramide-induced caspase-2 and caspase-8 activation [11]. Because cathepsin D acts upstream of caspase-8 but downstream of caspase-2, we next examined whether caspase-2 and GSK-3β caused lysosomal destabilization. Using AO staining followed by flow cytometric analysis, we found that both the caspase-2 inhibitor z-VDVAD-fmk (10 μM) and the GSK-3β inhibitor LiCl (10 mM) blocked C_2_-ceramide (25 μM) or etoposide (50 μM)-induced LMP in 10I cells (Fig 4A). In addition, confocal microscopic observation confirmed that treating cells with z-VDVAD-fmk or LiCl resulted in a blockade of cathepsin D re-localization from lysosomes to the cytoplasm in C_2_-ceramide- or etoposide-treated A549 cells (Fig 4B). To further confirm the effects of caspase-2 and GSK-3β, a lentiviral-based short hairpin RNA (shRNA) approach was performed. Western blot analysis showed the silencing of caspase-2 (Fig 4C, left top) or GSK-3β (Fig 4C, left bottom) expression in 10I cells treated with specific shRNAs. Caspase-2- (Fig 4C, middle) or GSK-3β-silenced cells (Fig 4C, right) were partially defective in C_2_-ceramide- or etoposide-induced LMP as well as cell apoptosis. These results demonstrate that both ceramide and etoposide can cause GSK-3β- and caspase-2-regulated LMP, cathepsin D re-localization, and cell apoptosis.

### Mcl-1 destabilization is regulated by GSK-3β, caspase-2, and the proteasome prior to LMP, cathepsin D re-localization, and mitochondrial apoptosis

**Fig 5:**
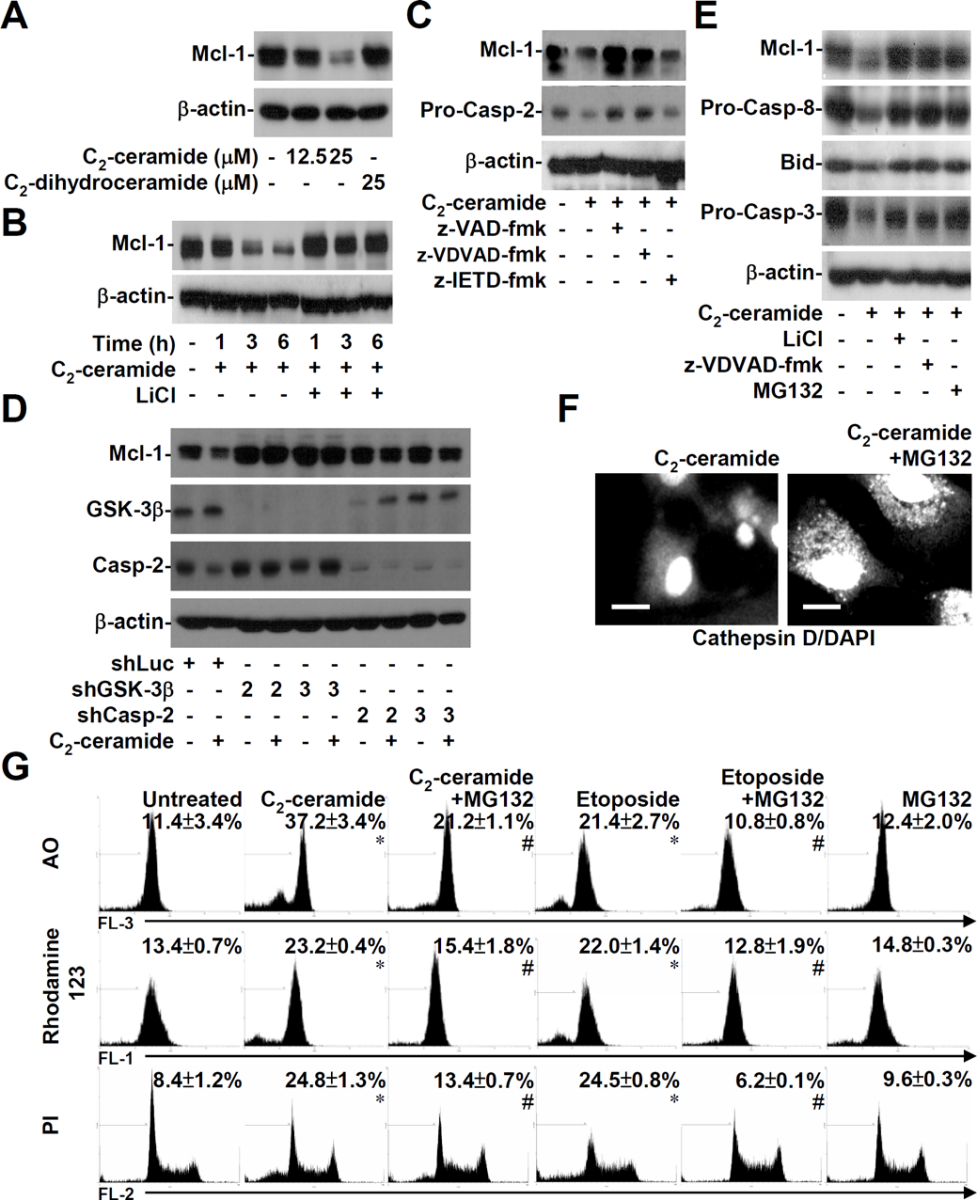
Ceramide induces GSK-3β-, caspase-2-, and proteasome-regulated Mcl-1 degradation followed by lysosomal-mitochondrial apoptosis. (A) We used Western blotting to determine the expression of Mcl-1 in 10I cells treated with different doses of C_2_-ceramide. C_2_-dihydroceramide was used as a negative control. (B) 10I cells were treated with 25 μM C_2_-ceramide in the presence or absence of 10 mM LiCl for the indicated time periods. We used Western blotting to determine the expression of Mcl-1. (C, D, and E) We used Western blotting to determine Mcl-1 expression and the activation of caspase-2, caspase-8, Bid, and caspase-3 in 25 μM C_2_-ceramide-treated 10I cells with or without z-VAD-fmk (10 μM), z-VDVAD-fmk (10 μM), z-IETD-fmk (10 μM), LiCl (10 mM), or the proteasome inhibitor MG132 (25 μM) for 6 h. 10I cells were transfected with shRNAs against GSK-3β (shGSK-3β) or caspase-2 (shCasp-2) or a negative-control shRNA (shLuc). The expression of Mcl-1 in ceramide-treated transfected cells was detected using Western blot analysis. β-actin served as an internal control. (F) A549 cells were treated with 25 μM C_2_-ceramide for 24 h in the presence or absence of 25 μM MG132. The translocation of cathepsin D was determined using a cathepsin D-specific antibody followed by an Alexa Fluor 488-labeled secondary antibody and DAPI nuclear staining. The scale bar is 10 μm. (G) 10I cells were treated with 25 μM C_2_-ceramide for 6 h in the presence or absence of 25 μM MG132. Using AO (top), rhodamine 123 (middle), and PI (bottom) staining, respectively, followed by flow cytometric analysis, the percentages of cells with LMP, MTP reduction, and apoptosis are shown (means ± S.D. of three individual experiments). A representative histogram is shown. *, *P* < 0.05 compared with the untreated group. #, *P* < 0.05 compared with the ceramide-treated or etoposide-treated group.

During cell apoptosis, activated GSK-3β causes Mcl-1 down-regulation through a mechanism involving phosphorylation followed by proteasome-mediated degradation [15]. Western blotting showed that C_2_-ceramide, but not C_2_-dihydroceramide, caused Mcl-1 destabilization in a dose-dependent manner (Fig 5A). Furthermore, C_2_-ceramide (25 μM) induced the time-dependent destabilization of Mcl-1 in a GSK-3β-dependent manner (Fig 5B). In addition to GSK-3β, caspases are also involved in Mcl-1 destabilization [17–19]. We showed that inhibiting caspase-2 but not caspase-8 decreased Mcl-1 destabilization in C_2_-ceramide-treated 10I cells (Fig 5C). C_2_-ceramide-induced Mcl-1 destabilization was also defective in both GSK-3β- and caspase-2-silenced cells (Fig 5D). These results demonstrate that activation of GSK-3β and caspase-2 mediate Mcl-1 destabilization in ceramide-treated apoptotic cells. To further verify the important role of Mcl-1 destabilization for ceramide-induced lysosomal destabilization and mitochondrial apoptosis, treatment with MG132 (25 μM), a proteasome inhibitor, blocked C_2_-ceramide-induced Mcl-1 destabilization and the activation of caspase-8, Bid, and caspase-3 (Fig 5E). Further results showed that MG132 blocked the re-localization of lysosomal cathepsin D to the cytoplasm in C_2_-ceramide-treated A549 cells (Fig 5F). Following AO-, rhodamine 123-, and PI-based staining and flow cytometric analysis, results showed that MG132 blocked C_2_-ceramide- or etoposide-induced LMP (Fig 5G, top), MTP reduction (Fig 5G, middle) and apoptosis (Fig 5G, bottom). These results show that Mcl-1 destabilization, which is regulated by GSK-3β, caspase-2, and the proteasome, occurs prior to ceramide- or etoposide-induced LMP, cathepsin D re-localization, and mitochondrial apoptosis.

### Lysosomal Mcl-1 is required for lysosomal stabilization

**Fig 6:**
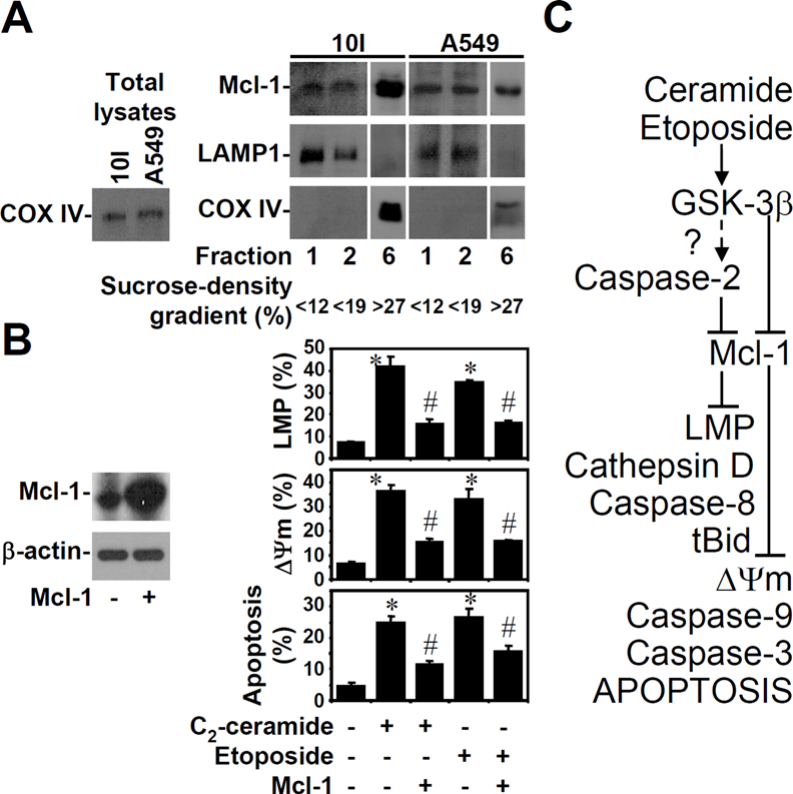
Mcl-1 expression is localized to the lysosomes, and forced Mcl-1 expression prevents ceramide- or etoposide-induced lysosomal-mitochondrial apoptosis. (A) Lysosomes and mitochondria were first isolated from untreated 10I or A549 cells. We used Western blotting to determine the expression of Mcl-1 in the different fractions of lysosome extracts. LAMP-1 and COX IV were detected as lysosomal and mitochondrial markers, respectively. Fractions obtained from sucrose-density gradient are lysosomes (<12% or <19%) and mitochondria (>27%). Total cell lysates were used as the positive control. (B) overexpression of human Mcl-1 in A549 cells. Mcl-1 expression was detected by Western blotting (top). β-actin served as an internal control. Cells treated with ceramide (25 μM) or etoposide (50 μM) for 24 h were stained with AO (second), rhodamine 123 (third), and PI (bottom), followed by flow cytometric analysis. The percentages of cells with LMP, MTP reduction, and apoptosis are shown (means ± S.D. of three individual experiments). *, *P* < 0.05 compared with the untreated group. #, *P* < 0.05 compared with the ceramide-treated or etoposide-treated group. (C) A schematic diagram of the pro-apoptotic role of GSK-3β in ceramide- or etoposide-induced lysosomal-mitochondrial axis-mediated apoptosis.

To further confirm the specific expression of Mcl-1, lysosomes were isolated from untreated 10I or A549 cells. Western blot analysis demonstrated the presence of Mcl-1 in mitochondrial extracts and in lysosomal extracts with no detectable mitochondrial contamination (Fig 6A). To examine the crucial role of Mcl-1 for lysosomal stabilization, artificially forced expression of Mcl-1 in A549 cells was shown by Western blotting (Fig 6B, left). Using AO, rhodamine 123, and PI staining, respectively, results demonstrated that Mcl-1 overexpression caused resistance in response to C_2_-ceramide (25 μM)- or etoposide (50 μM)-induced LMP, MTP reduction, and apoptosis (Fig 6B, right). These findings demonstrate that Mcl-1 is also critical for the maintenance on lysosomal stability, which protects cells from ceramide- or etoposide-induced apoptosis.

## Discussion

Ceramide or etoposide causes sequential activation of caspase-2 and caspase-8 upstream of mitochondrial apoptosis [24]. GSK-3β is a key regulator of these processes [11,13], and it is of particular interest to know whether there is cross-talk between GSK-3β and caspase-2 before caspase-8 activation. In the present study, as summarized in Fig 6C, we first verified that ceramide- or etoposide-induced lysosomal destabilization is regulated by GSK-3β, caspase-2, and Mcl-1. GSK-3β acts upstream of caspase-2 and synergistically with caspase-2 to induce lysosomal-mitochondrial damage following Mcl-1 destabilization. The mechanisms that underlie GSK-3β-regulated caspase-2 activation and caspase-2-induced Mcl-1 destabilization followed by LMP induction remain unclear. We further demonstrated that lysosomal cathepsin D acts upstream of caspase-8 and Bid activation before mitochondrial damage. Our results showed that Mcl-1 is essential for lysosomal membrane stabilization by inactivating LMP and cathepsin D re-localization.

Ceramide has been recognized as a second messenger for various apoptotic stimuli [21,22]. We previously demonstrated that ceramide and etoposide might induce mitochondrial apoptosis [22,24,25]. In the present study, using inhibitor and siRNA strategies, we showed that lysosomal cathepsin D is the common effector in ceramide- or etoposide-induced apoptotic signaling. The pro-apoptotic role of cathepsin D remains controversial, although the dependence on cathepsin D has been shown with a variety of apoptotic stimuli [8]. However, in stress-induced fibroblast cell apoptosis, which includes anticancer agents, irradiation, CD95, TNF-α, and ceramide, a cathepsin D-independent pathway has also been reported [28]. Therefore, cathepsin D-induced apoptosis depends on the cell type and different stimuli.

In the present study, we first verified the involvement of the lysosomal-mitochondrial axis in ceramide- or etoposide-induced apoptosis. Our results show that cathepsin D acts downstream of caspase-2 but upstream of caspase-8 before mitochondrial damage. Studies have suggested a possible mechanism of cathepsin D-mediated caspase-8 activation [10,29]. Indeed, caspase-8 and a variety of pro-apoptotic proteins, such as Bid and Bax, are also substrates of cathepsin D [8,10,29]. Consistent with these findings, we showed that cathepsin D was activated upstream of caspase-8 and Bid. Therefore, we hypothesize that ceramide or etoposide causes caspase-2 activation and then induces LMP followed by cathepsin D-mediated caspase-8 and Bid activation before mitochondrial apoptosis. Indeed, our current studies demonstrate that GSK-3β mediates ER stress-induced lysosomal apoptosis in leukemia involving caspase-2-induced LMP and cathepsin B relocation, which result in caspase-8 and -3 activation [30]. Furthermore, anesthetic propofol treatment induces GSK-3β-mediated LMP followed by cathepsin B-regulated mitochondrial apoptosis [31]. Combining the results of this study, an essential role of GSK-3β is therefore showed in lysosomal/mitochondrial apoptosis.

Ceramide regulates LMP partly by targeting cathepsin D directly [32]. In general, stress-induced LMP results from the activation of acid sphingomyelinase and ceramide generation, which binds and activates cathepsin D by autocatalytic processing [32,33]. Holman *et al*. [32,33] reported that inhibiting ceramidase results in increased ceramide levels in lysosomes, which then triggers apoptosis in prostate cancer cells. Therefore, lysosomes are potential targets for anticancer therapy [1,34]. We provide the first evidence to show that exogenous treatment with ceramide also causes LMP; however, it is unclear whether the acid sphingomyelinase-mediated pathway is required for the induction of LMP followed by activation and/or cytosolic translocation of cathepsin D.

In TNF-α-induced destabilization of the lysosome, caspase-8 is an effector for LMP [5]. However, our results showed that inhibiting cathepsin D blocked the activation of caspase-8 but not caspase-2. In contrast to the effects elicited by TNF-α, we demonstrated that the activation of caspase-2, but not caspase-8, was required for ceramide- or etoposide-induced LMP and cathepsin D translocation. This is the first report that caspase-2 acts upstream of not only the mitochondrial but also the lysosomal pathway in apoptosis.

Using inhibitors and shRNA, we provided evidence that GSK-3β partly regulates LMP and cathepsin D re-localization. The pro-apoptotic role of GSK-3β has been demonstrated previously [11]. During ceramide- or etoposide-induced apoptosis, activated PP2A induces GSK-3β activation [11]. PP2A is thought to act upstream of ceramide-induced LMP. Furthermore, GSK-3β is involved in diverse cellular responses because of its enzymatic activities on a broad range of downstream substrates, such as translation initiation factor 2B, β-catenin, p21^Cip1^, p53, Bax, and Mcl-1 [14]. However, although we previously showed that GSK-3β regulates ceramide-activated caspase-2 [11], the underlying mechanism remains unclear.

We further showed that GSK-3β and caspase-2 synergistically induced Mcl-1 destabilization followed by LMP. Consistent with previous studies [15,16], ceramide caused GSK-3β-regulated Mcl-1 destabilization through a proteasome-related mechanism. Inhibiting proteasomal function can prevent ceramide- or etoposide-induced lysosomal-mitochondrial axis-mediated apoptosis. Consistent with previous studies [17–19,35], in addition to the proteasome, we showed that caspase-2, similar to caspase-9 and csapase-3, mediated Mcl-1 destabilization.

Expression of Bcl-xL is known to be found in lysosomes, and Bcl-xL-overexpressing cells are protected from palmitate-induced LMP, as well as apoptosis [7]. Our studies showed that forced expression of Mcl-1 blocked LMP. Until now, it has remained unclear how Mcl-1 contributes to lysosomal stabilization because the regulation of Mcl-1 in lysosomes in response to stress is unknown. Generally, Mcl-1 inhibits the activation of Bid and Bax [35–37], which are both triggers of LMP [5–7], as well as MTP reduction [8]. Furthermore, Bax is positively regulated by PP2A and GSK-3β [5–7]. Caspase-2 has been demonstrated to be important for Bid and Bax activation [38–41]. We provide the first evidence to show the localization of Mcl-1 in lysosomes that may protect LMP from ceramide or etoposide stimulation. We hypothesize that Mcl-1 is the target of ceramide-activated GSK-3β and caspase-2 in the lysosomal-mitochondrial apoptotic pathway.

## Conclusions

We show that GSK-3β may facilitate caspase-2 activation through an unknown mechanism. Furthermore, GSK-3β causes Mcl-1 destabilization to induce the cathepsin D-mediated activation of caspase-8 and Bid in ceramide- or etoposide-induced apoptosis. Thus, the induction of lysosomal-mitochondrial axis-mediated apoptosis may underlie anticancer strategies involving ceramide or etoposide.
